# Sex difference in epigenomic instability during human aging

**DOI:** 10.18632/aging.204199

**Published:** 2022-08-01

**Authors:** Qihua Tan, Jonas Mengel-From, Kaare Christensen

**Affiliations:** 1 Epidemiology and Biostatistics, Department of Public Health, University of Southern Denmark, Odense, Denmark

**Keywords:** sex difference, epigenomic instability, DNA methylation, twins

Age-related change in the level of DNA methylation (DNAm) has been intensively investigated by epigenome-wide association studies (EWAS) performed on different human populations mainly using blood samples, showing a predominant pattern of decreased CpG methylation with increasing age although some methylated CpG sites also increase with age, but no significant sex-dependent pattern has been revealed [1, 2], except for a limited number of sites within genes implicated in sexually dimorphic traits [3]. The mean level of DNAm changing over age only reflects the general age trend or rate of change in the epigenetic regulation likely driven by non-stochastic or deterministic biological factors. Instead of examining the levels of DNAm change with age, which is estimated as the age-specific mean across individuals and over ages cross-sectionally, one could also focus on another important metric in describing a variable, that is the variance or variation of DNAm. This can be biologically meaningful in epigenetic studies because site-specific variation in DNAm among individuals of the same age could reflect how stable or instable the aging DNA methylome is. In this regard, the use of monozygotic (MZ) or identical twins enables controlling the genetic regulation of DNAm by, for example, the methylation quantitative trait loci or meQTLs. As an early example, Fraga et al. [4] reported larger difference in DNAm in a 50-year-old MZ twin pair when compared with that in a 3-year-old MZ twin pair, suggesting that both external (individual lifestyle, behavior, specific environmental exposure, etc) and internal (defect in maintaining epigenomic and genomic stabilities) factors affect the epigenome during the aging process.

Taking advantage of a large collection of genome-scale DNAm data on MZ twin pairs across different ages, we have recently performed an EWAS on age associated DNAm instability [5]. The large number of available twin samples allowed sex-stratified analysis to reveal sex difference in DNAm variability with age, without imposing any a priory assumption of the role sex may have. By fitting intra-pair DNAm difference as a function of age, more than three thousand CpG sites were found displaying age-dependent increase in DNAm variability with genome-wide significance in male twins but variability of these CpG sites were not age-dependent in female twins even though the same sample size and age distribution were used. Also, in both males and females more CpGs increased than decreased in intra-pair DNAm difference with age, although the pattern in females is only slightly seen. The same age pattern was confirmed in an independent younger cohort of MZ twin pairs, which also replicated 37% of the identified significant CpGs in male twins. These data led to the assumption that the DNA methylome in whole blood from men undergoes a striking epigenetic drift driven by non-genetic factors (environmental and stochastics) during the aging process. Interestingly, significantly variable methylation at CpG sites with age are mostly linked to genes functionally belonging to pathways related to cancer, a disease that constitutes one of the major causes of death worldwide.

For the first time, it was demonstrated that, the CpG sites exhibiting increased variability with age in men have been associated with survival. Here the twins with surplus hypermethylation tend to survive longer than their co-twins [5]. The observations may suggest that maintaining epigenetic stability during aging benefits us humans with a better chance to live a longer life, while a loss of epigenetic control could lead to an increased risk of death or a shorter lifespan. Of course, here comes an important issue of causality, i.e. is the observed change in DNAm variability the cause of aging or the consequence of aging (response to aging)? Future mathematical modeling and causal experimental designs should help to address this assumption.

Due to structural difference in the sex chromosomes between males and females, the sex chromosomes have been routinely ignored in current EWASs. One important topic remains to be explored is if X-chromosome inactivation (XCI) unique in women is an advantage for maintaining epigenetic stability. Chaligné et al. [6] reported that the inactive X-chromosome is epigenetically unstable and transcriptionally contributes to the development of breast cancer in females. On the other hand, women have the advantage of a back-up X-chromosome that may buffer DNA alterations and thus potentially avoid harming, e.g. hematopoietic stem cells [7]. With abundant DNAm data on the sex chromosomes available, strategic modeling that considers sex differences is called for in order to examine the roles of sex chromosomes in epigenetic stability during aging and their contribution to all-cause mortality.

In the literature of aging, sex difference in genomic instability has been reported in multiple studies. For example, chromosomal abnormalities increase with age, with the prevalence of age-related mosaic abnormalities being greater in males than in females on autosomes as well as sex chromosomes [8]. In cancers, age-adjusted mutation load is greater in males than in females. Furthermore, the somatic mutation accumulation was observed to start a decade earlier in males than in females [8]. These patterns are consistent with sex differences in age-adjusted incidence of cancer. In summary, genomic and epigenomic instabilities display a consistent pattern of sex differences which could help to explain the survival advantage in females.
